# Aggressiveness and emotion dysregulation among adolescents first degree relatives of schizophrenia patients

**DOI:** 10.1192/j.eurpsy.2023.2267

**Published:** 2023-07-19

**Authors:** F. Ghrissi, F. Fekih-romdhane, M. Stambouli, B. Abassi, M. Cheour

**Affiliations:** psychiatry department E, Razi Hospital, Mannouba, Tunisia

## Abstract

**Introduction:**

Schizophrenia is a severe debilitating condition, with elevated level of aggressiveness reaching 33% in a large sample of patients. Unaffected biological relatives of schizophrenia patients share similar though less severe neurocognitive and behavioral abnormalities seen in their affected relatives. Recent findings demonstrates that first degree relatives of schizophrenia patients are at increased risk of violence and aggressive behavior, especially during adolescence, with poor outcome. Besides, adolescents aged from 12 to 18 years old, may experience aversive and overwhelming emotions difficult to regulate due to immaturity of neuronal networks. There are evidence of an association of emotion dysregulation and violent conduct among youth. However, to our knowledge, studies among first degree relatives of psychotic patients were not performed.

**Objectives:**

The aim of this study was to evaluate the aggressiveness and emotion dysregulation among unaffected adolescents with fist degree family history of schizophrenia and to investigate the association linking these two entities.

**Methods:**

In this purpose wo conducted a cross sectional descriptive study in Razi hospital during three months: from July to September 2022. Unaffected adolescents aged 12 to 18 whom first-degree relatives were diagnosed with schizophrenia according to DSM-5 criteria were included. Adolescents with psychiatric conditions or medical affections associated with psychiatric presentation were not included. Sociodemographic data were collected on a preestablished questionnaire and the following scales were used: The Life History of Aggression LHA, an 11 items self-reported tool, in the Arabic version, The Aggression Questionnaire AQ which is a 29 items self-reported scale in Arabic version and the The Emotion Regulation Questionnaire (ERQ), a 10 items self-reported measure rated on a likert scale, in the validated Arabic version. Written informed consent was obtained from the legal tutor of each adolescent.

**Results:**

Results of this survey are ongoing.

**Conclusions:**

Results of this survey are ongoing.

**Disclosure of Interest:**

None Declared

